# Handicap theory is applied to females but not males in relation to mate choice in the stalk-eyed fly *Sphyracephala detrahens*

**DOI:** 10.1038/s41598-020-76649-3

**Published:** 2020-11-12

**Authors:** Koji Takeda, Tomoki Furuta, Masaki Hamada, Yo Sato, Kiichiro Taniguchi, Akihiro Tanizawa, Tomomasa Yagi, Takashi Adachi-Yamada

**Affiliations:** Department of Life Science, Faculty of Science, Gakushuin University, 1-5-1 Mejiro, Toshima-ku, Tokyo, 171-8588 Japan

**Keywords:** Evolution, Zoology

## Abstract

Handicap theory explains that exaggeratedly developed sexual traits become handicaps but serve as honest signals of quality. Because very weak signals are less likely to provide benefits than to simply incur costs, it is interesting to elucidate how sexual traits are generated and developed during evolution. Many stalk-eyed fly species belonging to tribe Diopsini exhibit marked sexual dimorphism in their eye spans, and males with larger eye spans have larger bodies and reproductive capacities, which are more advantageous in terms of contests between males and acceptance for mating by females. In this study, we investigated the role of eye span in a more primitive species, *Sphyracephala detrahens,* in tribe Sphyracephalini with less pronounced sexual dimorphism. Male-male, female-female, and male–female pairs showed similar contests influenced by eye span, which was correlated with nutrition and reproductive ability in both sexes. During mating, males did not distinguish between sexes and chose individuals with larger eye spans, whereas females did not choose males. However, males with larger eye spans copulated repeatedly. These results indicate that, in this species, eye span with a small sexual difference does not function in sex recognition but affects contest and reproductive outcomes, suggesting the primitive state of sexual dimorphism.

## Introduction

Two contradictory explanations for the evolution of extremely developed sexual traits stimulating visual, auditory, and olfactory sensors have historically received the most attention: the runaway hypothesis^1^ and handicap theory^2–4^. According to the runaway hypothesis, when a sexual trait functions in sexual recognition, larger values of the trait provide stronger signals that are more easily recognized, leading to evolution of the trait. However, because large sexual traits are costly and not necessarily adaptive, the advantages for the opposite sex are likely to be small, except in the case of genetic factors that result in superficially attractive offspring^5^. In contrast, handicap theory postulates that expressing extremely developed secondary sexual characteristics (i.e., sending a strong signal) is a handicap and can be achieved only by high-quality individuals. In this case, the size of the trait becomes an honest signal of quality, and because the biological trait is likely to have a genetic basis, the merits gained from the signal benefit both sexes and offspring in various aspects^6^. Moreover, prominent sexual traits often exhibit strong positive allometry with body size^7^, and sexual traits of individuals raised in better nutritional environments tend to be more apparent^8^.

Although this argument leads us to predict that sexual dimorphism will evolve to function in mate preference, this function is unlikely to be a driving force until sexual dimorphism is already somewhat pronounced. This is because it is difficult to recognize a slight difference between sexes that has just arisen. The “dimorphism niche hypothesis” proposes that dimorphism in secondary sexual characteristics evolves irrespective of sex recognition^9–11^ and that the difference arises because males and females originally possessed different traits in which to invest. For example, in many insects, the abdomen of females is more swollen and rounder than that of males, probably because of the large space required for egg maturation and thus greater investment in offspring^12,13^.

Stalk-eyed flies are dipteran insects with sexual dimorphism in the highly developed eye stalk, the role of which has been well studied^14,15^. A representative species is *Cyrtodiopsis dalmanni,* which belongs to tribe Diopsini. In ritualized contests based on eye spans, males face other males, and the longer-eyed individual wins and holds the territory. Although this morphology is known to bring about physical handicaps, such as impaired flight ability^16,17^, eye span shows positive allometry with body length and nutrient intake^18^ and correlates with aspects of fertility, such as accessory gland size^19^. Because females prefer males with longer eye spans^20^, handicap theory can be applied to this species.

In this study, we used *Sphyracephala detrahens*, the only stalk-eyed fly species inhabiting Japan, which belongs to tribe Sphyracephalini, a sister group of tribe Diopsini. Sphyracephalini is a more primitive group than Diopsini and exhibits weak sexual dimorphism^21^. Therefore, it was expected that this species could be a suitable material for investigating the origin of sexual dimorphism in stalk-eyed flies. For this purpose, it was necessary to test the following four hypotheses for properly applying the handicap theory to this species: (1) Eye span acts as an honest signal to inform opponents of fighting capacity. (2) Eye span functions as an honest signal to inform the opposite sex of reproductive capacity. (3) Males cannot distinguish between sexes. (4) Eye stalk is a costly organ. All of these hypotheses have been proven to be correct as described below.

In our study, a traditional experiment involving a simple contest between individuals was carried out by placing two individuals in an arena. However, in an experiment to assess mate preference and sexual recognition, we implemented a new method as follows: to confirm whether a male (or female) having a longer eye span was chosen for mating, one female (or one male) and two males (or two females) having different eye spans were allowed to coexist in one arena.

In an experiment designed to investigate whether males can recognize sex, we utilized two special characteristics of this species: males frequently pseudo-copulate with other males, and individuals with longer eye spans are preferred as mating partners (as described below). First, we focused on the mating behaviour of medial-eyed males among three coexisting males with different eye spans. After confirming that this male selectively pseudo-copulated with a longer-eyed male, the shorter-eyed male was replaced with the shortest-eyed female, and behavioural observation was continued. We expected that if a medial-eyed male reluctantly pseudo-copulated with another male and could recognize sex when a female was present, he would switch to copulate with the female, even if that female had the shortest eye stalks. In contrast, we expected that if the medial-eyed male continued to pseudo-copulate with the longer-eyed male despite the presence of the female, eye span would be used as a more valuable signal when selecting the mating partner, and sex recognition would not occur. However, this method has the disadvantage that the females available for selection have the smallest eye span among the four flies. To select males and females in a fairer way, a mixture of medial-eyed males with long- and short-eyed males/females was prepared, and the behaviour of medial-eyed males was the focus. Based on these experiments, it was inferred that, despite the slight sexual dimorphism, the difference was not used for sex recognition. If the “missed opportunity cost” due to a lack of sex recognition is less than half the cost of correctly discriminating sex by utilizing slight sexual differences, such non-discrimination will be maintained.

Taking these results together, the evolutionary origin of sexual dimorphism in the stalk-eyed fly is not considered to be related to sex recognition but based on sexual differences in the value of winning contests, subtracted by male preference for long-eyed females. In addition, handicap theory, which has been applied to the eye-span signal in relation to male mating behaviour in Diopsini flies, can be applied only to female eye span in this primitive state.

## Results

### Mild sexual dimorphism in *S. detrahens*

**Figure 1:**
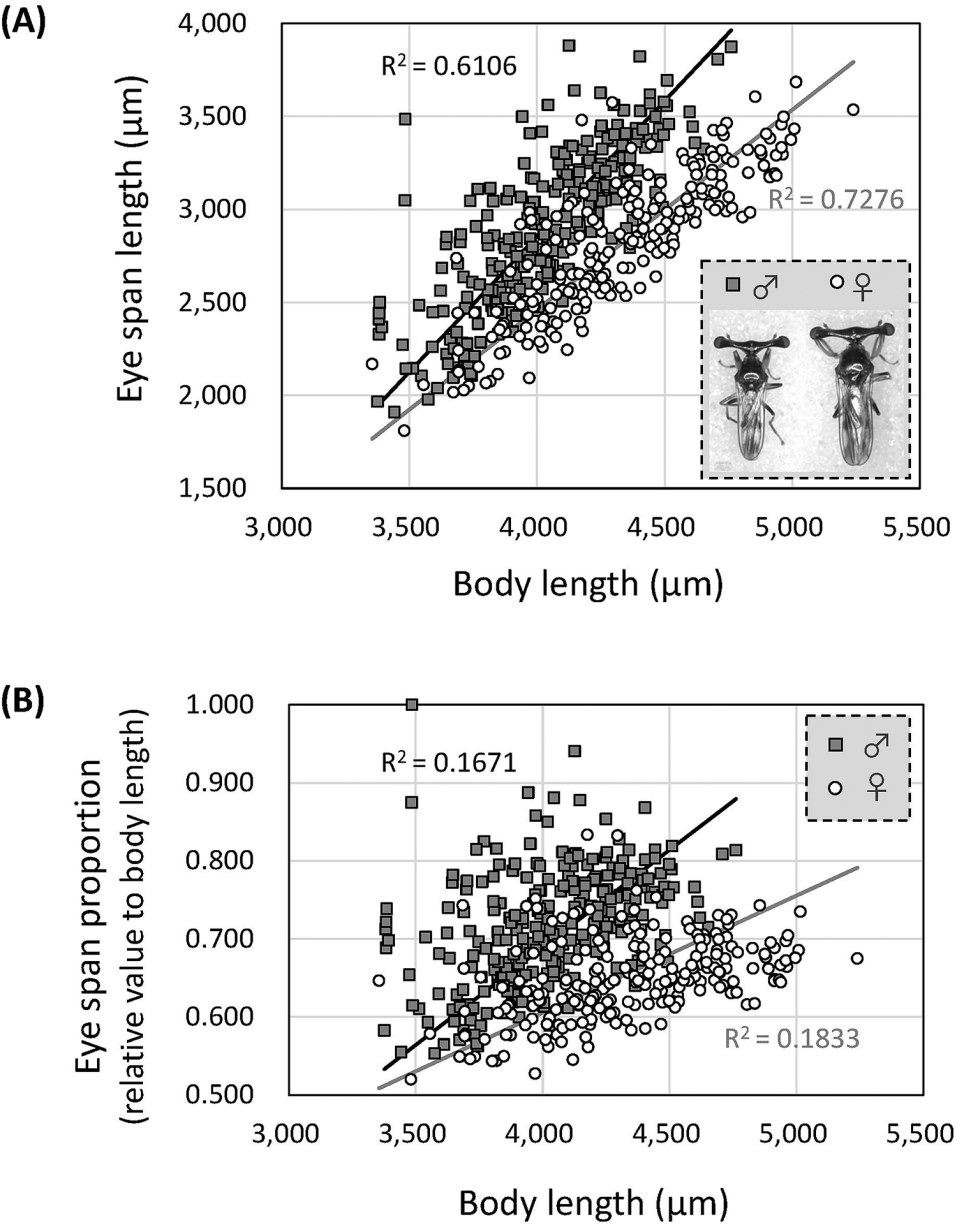
Mild sexual dimorphism in *S. detrahens* in terms of the proportion of eye span relative to body length. (**A**) Relationship between body length and eye span in 486 individuals (262 males and 224 females) collected from the natural habitat. Approximation lines were drawn by linear approximation expressed with model 2 regression. Multiple coefficients of determination (R^2^) = 0.6106 (males) and 0.7276 (females). The P values from Student’s t-tests between males and females were 0.014 for eye span and 8.1 × 10^–22^ for body length, indicating significant differences. The P value from the analysis of covariance (ANCOVA) between slopes associated with males and females was 0.0010, indicating a significant difference. The inset shows male (left) and female (right) adults. The image was processed by Adobe Photoshop CC 2019 software (www.adobe.com). (**B**) Relationship between body length and relative eye span (eye span/body length). Approximation lines were drawn by linear approximation expressed with model 2 regression. R^2^ = 0.1671 (males) and 0.1833 (females). The P value from Student’s t-test of the difference in eye-span proportion between males and females was 9.8 × 10^–31^, indicating a significant difference.

Mild sexual dimorphism in the eye span of *S. detrahens* was previously reported^20^, but the details have not been well documented. Thus, we examined individuals collected from wild habitats. As shown in Fig. 1A, the distributions of eye spans and body length largely overlapped between males and females. Therefore, when focusing on one individual, it was difficult to distinguish males and females based on only these variables (Fig. 1A inset). However, because the slope of the approximation line, which represents relative growth between eye span and body length, differed greatly between males and females (P value from Student’s t-test = 0.014), mild sexual dimorphism could be recognized overall. Furthermore, the eye span proportion (ratio of eye span to body length) increased with increasing body length in both sexes, and the slope was significantly steeper in males than in females (Fig. 1B, P value from Student’s t-test = 9.8 × 10^–31^). The allometric slopes (least-squares regression slope for eye span to body length) in males and females were 1.14 ± 0.0564 (standard error, SE) and 0.917 ± 0.0377 (SE), respectively. The dimorphism index (= difference between male and female allometries) was 0.222. This value is slightly larger than that in *Sphyracephala beccarii* (0.197), a related species also known to show mild sexual dimorphism, and much lower than that in the highly dimorphic *C. dalmanni* (1.168)^22^. Taking these results together, there is a slight difference in resource allocation to the eye stalk between the sexes of *S. detrahens*.

### Hypothesis 1: Eye span acts as an honest signal to inform opponents of fighting capacity

As mentioned above, many past studies have shown that males of stalk-eyed flies inform their opponents of their fighting capacity based on their eye spans, and individuals with larger eye spans win^14,23^. Therefore, in this species, we hypothesized and verified that eye span acts as an honest signal of fighting capacity that informs the opponent. Because contests between females and between males and females occur in this species, we investigated such contests similarly to male-male contests. We randomly combined individuals with various eye spans, measured the winning rates when a contest consisted of 10 or more games, and examined the correlations between the eye span ratios of both individuals and the winning rate.

**Figure 2:**
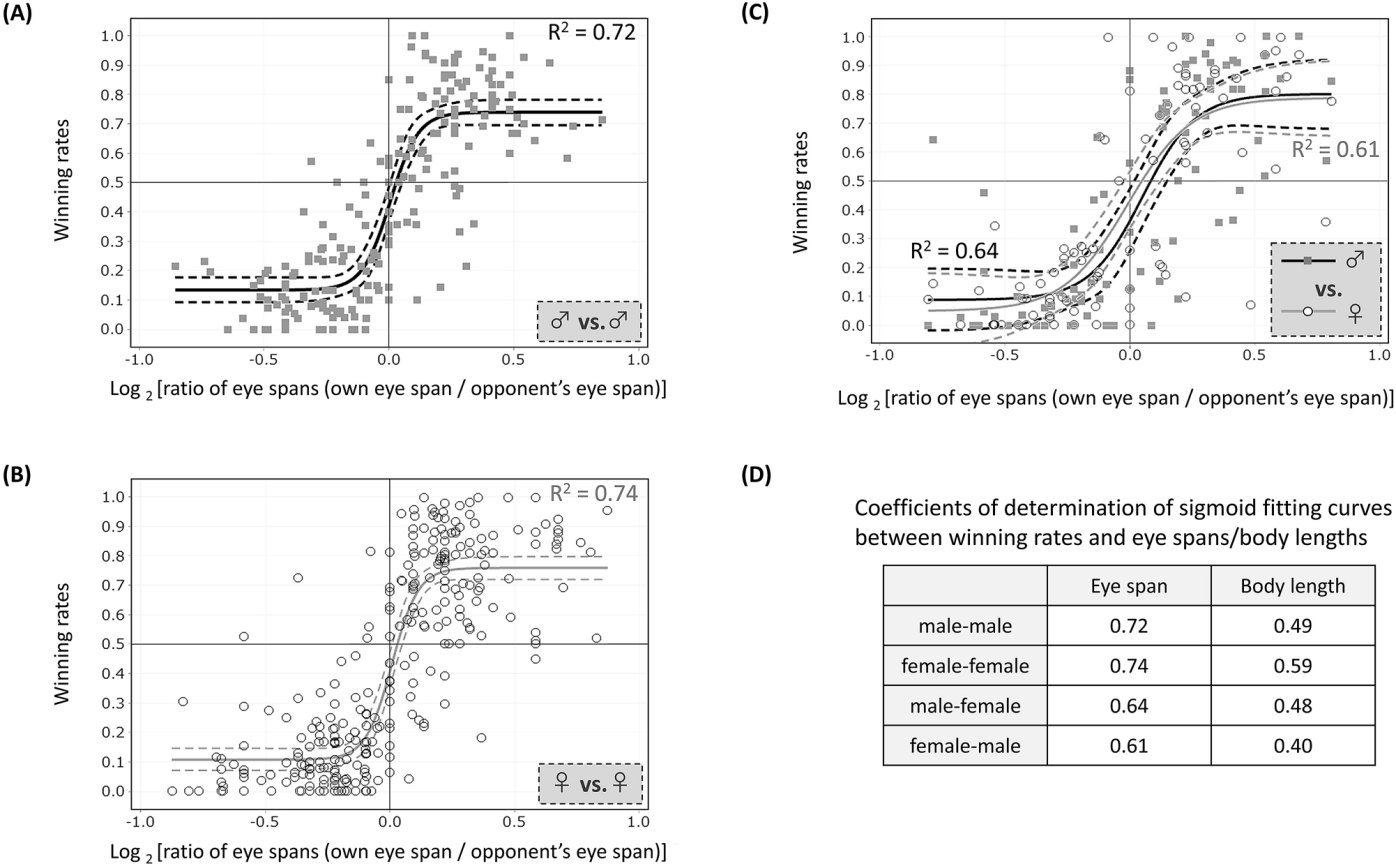
Flies with relatively long eye spans are likely to be winners in contests. Winning rates in 100 (**A**, male vs. male), 140 (**B**, female vs. female) and 88 (**C**, male vs. female) contests including more than 10 games for pairs of randomly chosen individuals with different eye-span lengths were measured and analysed in regard to their relationship with the eye-span ratio (own eye span/opponent’s eye span). Each datum point represents one player’s outcome in a contest on one pair of flies with different eye spans. A single contest generates two data points: one for long-eyed fly and the other for short-eyed fly. Because all the winning rates included draws in the denominator, the mean values in each sexual combination are less than 0.5. Only one data point in A, for which the eye-span ratio is 2.15, was excluded as an outlier. Solid lines were drawn by sigmoid approximation via the Gauss-Newton algorithm (Minitab 16, Minitab Inc.). R2 = 0.72 (**A**), 0.74 (**B**), 0.64 (males in **C**), and 0.61 (females in **C**). Broken lines indicate 95% confidence intervals. The P values of the three approximation lines are all less than 0.001, indicating significant fits. The P value from the ANCOVA of the datum point distribution among the three kinds of sexual combinations was 0.9469, indicating a non-significant difference.

Figure 2A shows the relationship between eye-span ratio and winning rate in contests between males. Contest winners were biased towards individuals with longer eye spans (82%, P value from Pearson’s Chi-square test = 4.0 × 10^–11^). Further, a player became more likely to win contests when his eye span was even slightly larger than his opponent’s, but the winning rate did not increase further as the eye span ratio increased. Furthermore, since the winning rate can never exceed 1, an approximation line was drawn by a sigmoid function (Supplementary Information 3). The coefficient of determination representing the correlation was 0.72. Similar striking correlations were also found in contests between females (Fig. 2B) and in contests between males and females (Fig. 2C), with winning rates for longer-eyed individuals of 85% (P value = 6.3 × 10^–18^) in female-female contents and 78% (P value = 5.4 × 10^–8^) in male–female contents and coefficients of determination of sigmoid fitting curves of 0.74 and 0.61-0.64, respectively. In the contests between males and females, neither sex was more likely to win. The fitted curves for these three kinds of sexual combinations were very similar and indistinguishable by covariance analysis. Based on these results, it appears that this species participates in contests in which eye span acts as an honest signal of fighting capacity that informs the opponent similarly in all three kinds of sexual combinations.

However, among the contests in which the difference between the eye spans of both individuals was small (less than 10%), the male-male contest displaying the winning rate of around average 41.9% (more than 31.9% and less than 51.9%) showed a higher proportion than contests of other sexual combinations (26% in male-male contents, 13% in female-female contests, and 14% in male–female contests). This might suggest a tendency for males to not readily give up during a game.

### Hypothesis 2: Eye span functions as an honest signal to inform the opposite sex of reproductive capacity

As mentioned above, in a previous study of stalk-eyed flies in another tribe, Diopsini, it was thought that eye span functioned as an honest signal of reproductive capacity that informed the opposite sex because males with larger eye spans had higher fertility and females gathered around such males^20^. Therefore, we hypothesized and verified this function in both sexes of *S. detrahens*.

**Figure 3:**
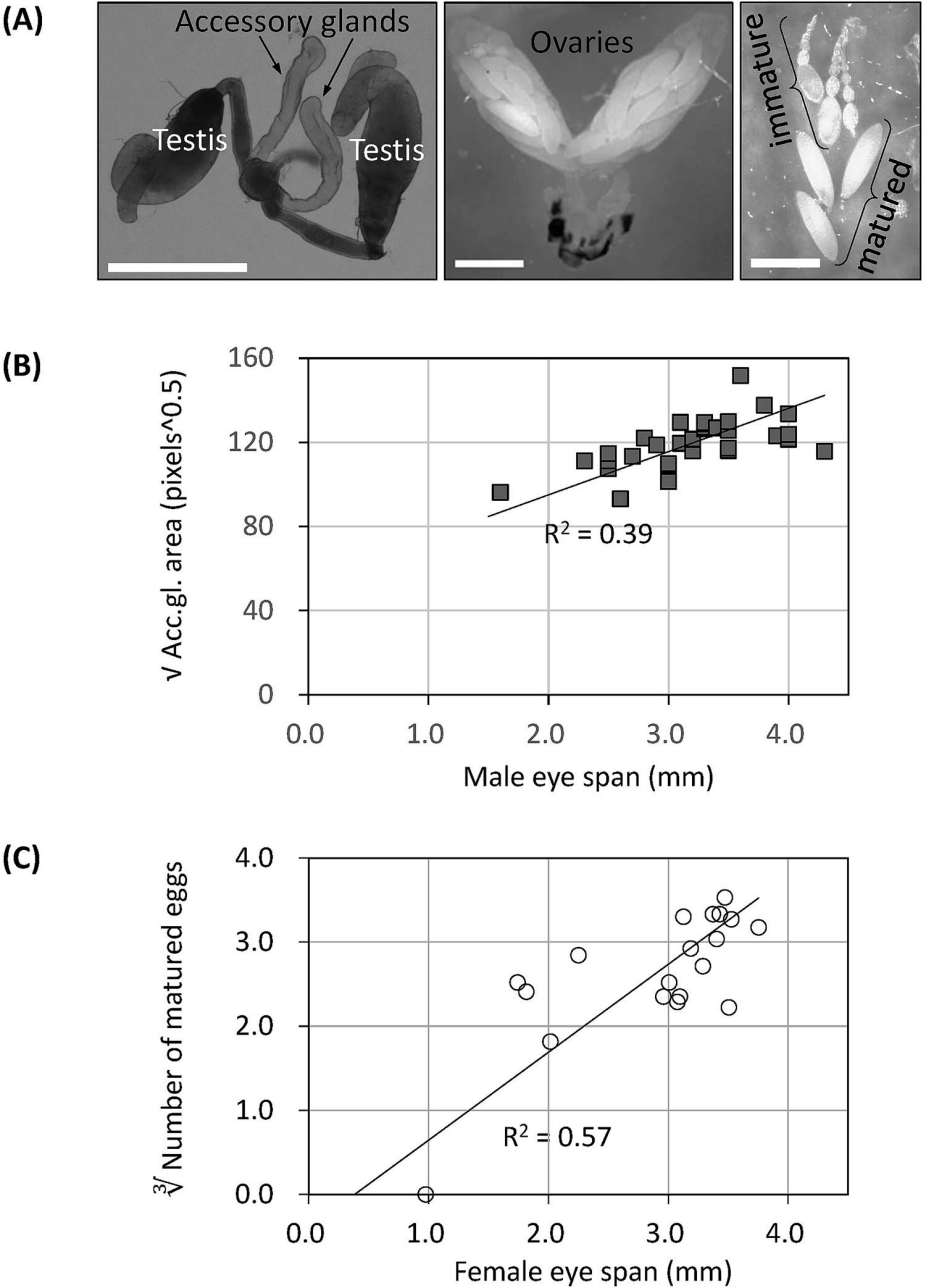
Relationship between eye span and reproductive organ size. (**A**) Internal reproductive organs of adult *S. detrahens*. Left: male accessory gland, middle: female ovaries, right: immature and mature eggs. Scale bars: 500 μm. The images were processed by Adobe Photoshop CC 2019 software (www.adobe.com). (**B**) The relationship between eye span and the square root of the surface area of the accessory gland was examined in 32 adult males. An approximation line was drawn by linear approximation expressed with model 2 regression. R^2^ = 0.37, P value = 0.000219. A moderate positive correlation between the two factors was found. (**C**) The relationship between eye span and the cube root of the number of mature eggs was examined in 19 adult females. An approximation line was drawn by linear approximation expressed with model 2 regression. R^2^ = 0.44, P value = 0.0018. A moderate positive correlation between the two factors was found.

First, we confirmed the correlation between eye span and reproductive capacity. The size of the male accessory gland (Fig. 3A left), which is known to reflect male reproductive capacity in various insects^19,24^, was measured, and its correlation with eye span was examined (coefficient of determination = 0.37, P value = 0.000219). Similarly, to examine the fertility of females, the number of mature eggs (Fig. 3C) in the ovary (Fig. 3B) was measured, and its correlation with eye span was examined (coefficient of determination = 0.44, P value = 0.0018). The results showed that eye span and reproductive capacity were correlated in both sexes, as shown in previous studies in other stalk-eyed fly species^15,19,25,26^.

**Figure 4:**
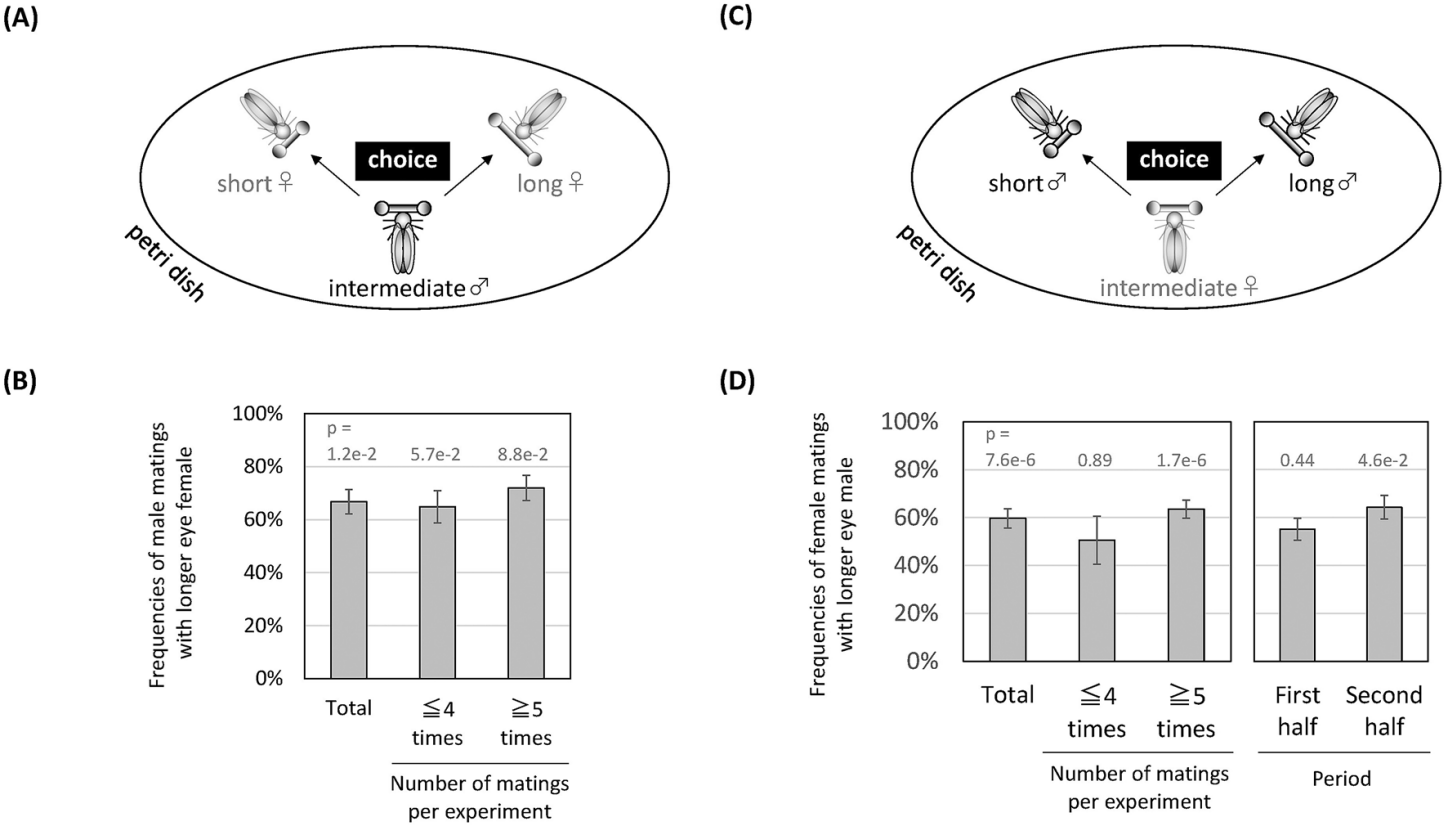
Mate choice in males and females in relation to eye span. (**A**) Experimental design. Two females with different eye spans and one male with an intermediate eye span were placed in a Petri dish and allowed to mate freely. (**B**) Results of 58 tests evaluating male choice of longer-eyed females. Frequencies are shown in percentages with standard errors and are categorized into three groups with respect to the number of matings (total, 4 times or fewer, and 5 times or more). P means P values according to Pearson’s Chi-square tests. (**C**) Experimental design. Two males with different eye-span lengths and one female with an intermediate eye-span were placed in a Petri dish and allowed to mate freely. (**D**) Results of 53 tests evaluating female choice of longer-eyed males. Frequencies are shown in percentages with standard errors. In the left graph, data are categorized into three groups with respect to the number of matings (total, 4 times or fewer, and 5 times or more). In the right graph, data are categorized into two groups with respect to the timing of mating during observation (first half and second half). P means P values according to Pearson’s Chi-square tests. The illustrations in (**A**) and (**C**) were processed by Microsoft PowerPoint 2007 software (www.microsoft.com).

Unless eye span affects the behaviour of signal receivers in a way that benefits the signaller, it will not be stabilized as a signal during evolution^27,28^. Therefore, we examined whether males or females preferred individuals of the opposite sex with larger eye spans. As described in the “Methods” section, we first placed two females with different eye spans and one male with an intermediate eye span in a Petri dish together and examined which female mated more (Fig. 4A). Males more frequently copulated with females with larger eye spans (Fig. 4B, 66.8%, P value from Pearson’s Chi-square test = 0.012), but the effect of the number of copulations on male mate choice was not clear. Similar male mate preference has also been reported in the dimorphic species *C. dalmanni*^29^. Next, for the mate preference of females, we reciprocally placed two males with different eye spans and one female with an intermediate eye span in a Petri dish together and examined which male mated more (Fig. 4C). Females copulated with males with larger eye spans at a high frequency (Fig. 4D left, 59.7%, P value from Pearson’s Chi-square test = 0.0000076), but the result was strongly influenced by the number of copulations and especially pronounced in cases of more than 5 copulations (Fig. 4D left). Furthermore, the tendency was strong in the second half of the observation period but not in the first half (Fig. 4D right). These findings suggest that males with larger eye spans can mate repeatedly in a short period of time, but females do not choose males with large eye spans, and males with small eye spans can mate only a few times. In fact, when observing mating behaviour, it appeared as though females were not given the opportunity to choose a male, as the male suddenly jumped over the female and began mating in the middle of the fighting with the female (Supplementary Information 1B). In addition, there was no clear correlation between mate choice and own eye span (coefficient of determination = 0.0003 in males and 0.0033 in females).

According to these results, female eye span functions as an honest signal for informing the male of the female's reproductive capacity, but whether male eye span is informative for the female is not known. Even if the male sends some signals, we do not know if he would benefit from doing so. This situation is different from that in sexually dimorphic stalk-eyed fly species in which males with larger eye spans are chosen by females. However, it has been reported that in the sexually monomorphic species *Cyrtodiopsis quinqueguttata*, females do not choose males based on eye span^20^.

### Hypothesis 3: Males cannot distinguish between sexes

If only males choose females properly, a question arises. This is because males of this species frequently pseudo-copulate with each other (Supplementary Information 1C), which is rare in the well-studied species *C. dalmanni*. Therefore, we hypothesized and verified that males of *S. detrahens* cannot discriminate between the sexes, although they have a preference for a certain eye span in the opponent.

**Figure 5:**
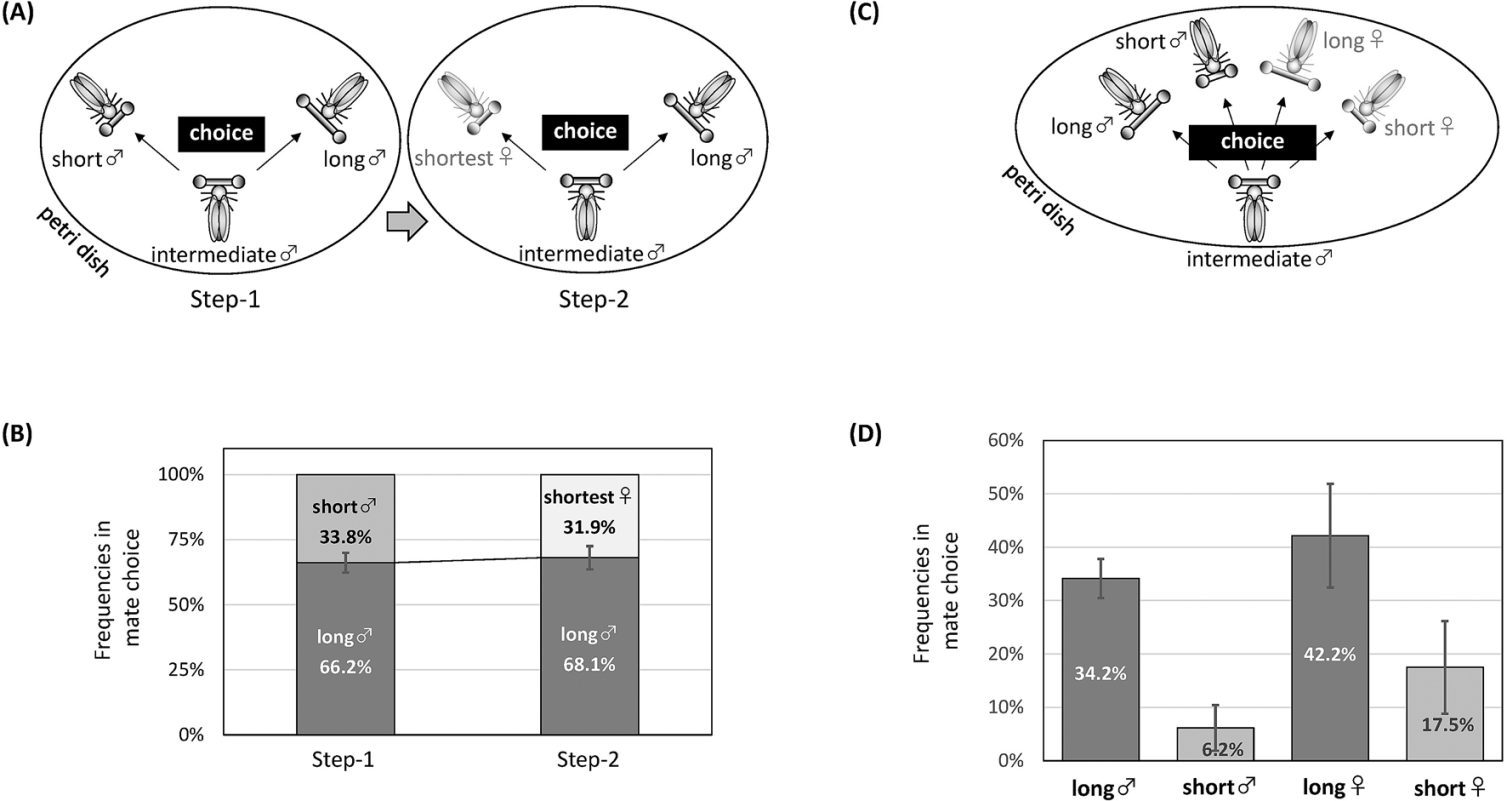
Inability of males to discriminate between sexes during mating behaviours. (**A**) Experimental design-1. After a certain time period over which male-male pseudo-copulation was observed (Step 1), the short-eyed male was replaced with the shortest-eyed female to continue the observation of mate choice (Step 2). N = 30. (**B**) Frequencies of mate choice of the intermediate-eyed male for each individual are indicated on each bar. (**C**) Experimental design-2. A fly group composed of three males (short, intermediate, and long eye spans) and two females (short and long eye spans) was prepared. In six fly groups, the intermediate males were focused on to observe their mate choice. (**D**) Average frequencies of mate choice of the intermediate males for each individual. The P values between individuals were 0.001 (long-eyed male vs. short-eyed male), 0.5 (long-eyed male vs. long-eyed female), and 0.1 (long-eyed female vs. short-eyed female). The illustrations in (**A**) and (**C**) were processed by Microsoft PowerPoint 2007 software (www.microsoft.com).

Three males with different eye spans were placed in a Petri dish, and the behaviour of males with intermediate eye spans was the focus (Fig. 5A). The males preferentially pseudo-copulated with males with larger eye spans (Fig. 5B left, 66%, P value from Pearson’s Chi-square test = 0.00785). After that, the male with the short eye span was replaced with a female with the shortest eye span, and the behaviour of the male with an intermediate eye span continued to be observed. We expected that if males could distinguish between sexes and selectively mate with females, the target of the male that preferred a longer-eyed male would change. However, the preference did not change (Fig. 5B right, 68%, P value from Pearson’s Chi-square test = 0.00076), suggesting that males cannot distinguish between sexes but prefer to copulate with individuals with larger eye spans.

However, one caveat is that in this experiment, the only female had the smallest eye span among the options. Females with the smallest eye span may be unlikely to be chosen because they are inferior to other individuals in terms of other characteristics, such as body length. Therefore, we tested the preference of the intermediate-eyed males when four males and females with different eye spans were provided as options (Fig. 5C). Males still chose their mating partners by prioritizing eye span and did not significantly distinguish between sexes (Fig. 5D).

### Hypothesis 4: Eye stalk is a costly organ

**Figure 6:**
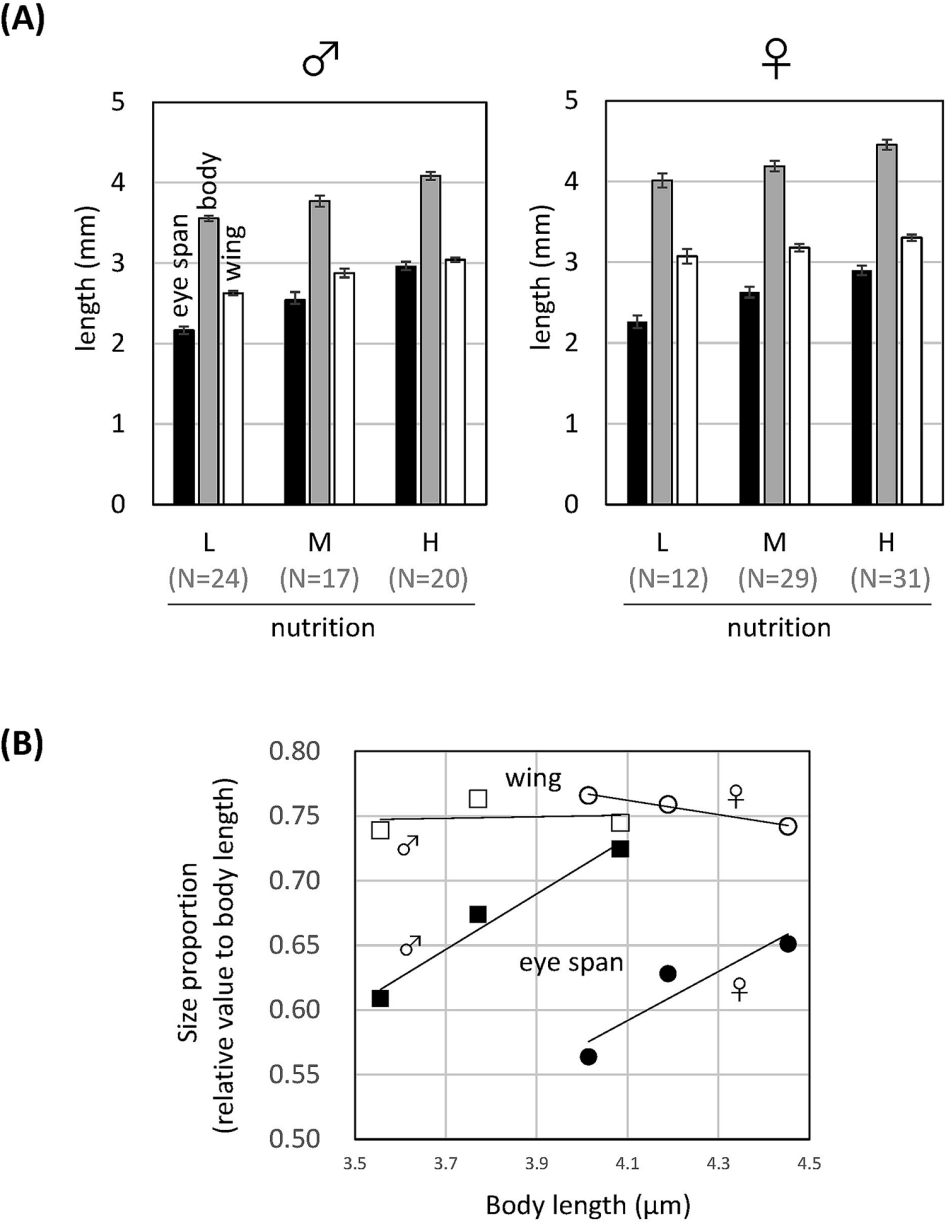
Effect of nutrition intake on body and body part sizes. (**A**) Lengths of the eye span, body, and wing of individuals raised under various nutritional conditions (L: low, M: medial, H: high). Left: male, right: female. The number of measured individuals (N) is shown in parentheses below the bars. (**B**) Proportions of eye span and wing length relative to body length under various nutritional conditions. Closed squares: male eye spans, closed squares: female eye spans, open squares: male wings, open circles: female wings.

According to the above results, the eye stalk has an advantage in contests for both males and females, while in females only, it may be a signal that conveys information to the opposite sex. If handicap theory, in which strong signals are produced only by high-quality individuals, can be applied to this situation, the eye stalk must be costly. Therefore, we bred individuals under various nutritional conditions and examined the length of the eye stalk (Fig. 6A).

In both sexes, eye span, body length, and wing length increased according to nutritional level, and the ratio of wing length to body length did not change very much, but the ratio of eye span to body length strongly increased (Fig. 6B). These results are consistent with the distributions of values in the natural population (Fig. 1B). These observations showed that, in this species, high nutritional intake is required for a large eye span, the formation of which incurs an impossible cost for individuals with low nutrient intake. Previous studies revealed that in *C. dalmanni*, which has strong sexual dimorphism, the nutritional dependence of eye stalk size is male-specific, but in *S. beccarii*, which has weak sexual dimorphism, it occurs in both males and females^30^.

## Discussion

### Factors for winning contests

In past studies of contests in sexually dimorphic stalk-eyed flies, males judged the ability of their opponents by using eye span as an index, while females utilized factors other than eye span^25,31^. However, in *S. detrahens*, a primitive species observed in this study, eye span was utilized similarly in male–female, female–male, and male–female contests. Although such indiscriminate fighting among all types of individuals seems incomprehensible and meaningless in the context of previous studies, it can be accepted as a rather consistent phenomenon since this species does not exhibit strong sexual dimorphism and cannot distinguish between sexes. For all three sexual combinations, we also examined and compared the correlation between body length and winning rate. In each case, eye span showed a stronger correlation with winning rate than did body length (Fig. 2D). Although we cannot confirm that eye span is the only direct cause of winning^25,31^, it at least partially determines the outcome of a contest because it is the most prominent visual signal when facing the opponent.

### Factors affecting mild sexual dimorphism

Why is the eye span proportion larger in males than in females, despite females clearly displaying it as a self-valuable intersexual signal? One possible reason is that the value of contests differs between males and females. Because the habitat of this species usually lacks nutritional resources, all individuals are thought to fight for space in order to secure food. In fact, if individuals are artificially fed excess food that makes fighting unnecessary, all the individuals quit fighting and gather in one place to concentrate on feeding (Supplementary Information 4). Therefore, in the natural state, males should secure a location with a nutritional resource that is easy for females to visit. While males can increase their offspring as they mate with new females in such a location, females may not benefit as much from remaining in the territory after mating as males because females will have more offspring once mated, similar to patterns in many other animals. Therefore, females do not fight as intensely as males, which is also reflected in the distribution of the winning rate, as mentioned above (Fig. 2). For this reason, there is a difference in the value of the required resources between males and females, which may explain why males receive more value from investing in the eye stalk. In addition, males with a large eye span have a high reproductive capacity and can repeatedly mate; thus, they can win in sperm competition. These features may explain the sexual dimorphism in this species without the need to invoke the runaway hypothesis or handicap theory regarding the signal sent to the opposite sex. However, as mentioned above, eye stalk formation obviously incurs a large cost because of nutrition investment, and it is an honest signal of reproductive capacity. Thus, handicap theory can be applied only to females that let males choose. Such behaviour may at least partly affect the female eye span. However, when focusing on a specific individual (not on a whole population), as some individuals produce a small number of mature eggs relative to their eye spans (lower right region of the approximation line in Fig. 3C), a positive correlation between eye span and the number of mature eggs can be expected as a whole. Although there is no evidence that female eye span is genetically controlled, there is also no evidence that it is determined only by environmental factors. As in many examples of animal organogenesis, we should regard female eye span as being controlled by both genetic and environmental factors. In the case of another tribe, Diopsini, the involvement of genetic factors in eye span variation has already been documented^32^. Eye stalk extension in males may serve as a pre-adaptation for the establishment of female mate preference after additional evolution and easier sexual recognition, which may represent a primitive state of sexual dimorphism in stalk-eyed flies.

### Reason why males do not distinguish between sexes

Is it not a disadvantage that these males do not distinguish between sexes? The pseudo-mating behaviour of males with other males consumes unnecessary energy, leading to a decrease in fitness. However, to avoid this cost, males need to acquire sex recognition ability, and this species, with a very slight sex difference in eye span on average, seems to incur another cost of discrimination. This can be regarded as a kind of life-history trade-off in which the cost can be lowered by choosing discrimination or non-discrimination^33^. Alternatively, it could be interpreted as a choice between speed and accuracy in sex recognition^34^. This is not uncommon among animals that do not distinguish between sexes due to the low cost of false mating, such as males of the European toad (*Bufo bufo*)^35^ and females of the orange chromide (*Etroplus maculatus*)^36^. In the case of this stalk-eyed fly, males and females are considered to be equal in frequency. Hence, if the cost generated by random trials of copulation without sex recognition is less than half the cost of identifying males and females, non-discrimination of sexes will be stabilized. The cost of pseudo-copulatory behaviour is regarded as a “missed opportunity cost”^37^ and is related to the ability to distinguish between correct and incorrect foods during foraging^34^.

### Application of the “erroneous courtship hypothesis” to contest and mating behaviours in *S. detrahens* in association with its poorly developed sexual traits

Mild sexual dimorphism in tribe Sphyracephalini has been recorded in Baltic amber fossils formed approximately 40–50 million years ago^38^. Thus, the present mild sexual dimorphism found in *S. detrahens* can be regarded as a result of evolution cessation unless unknown factors that drive fluctuations in eye span are considered. It is difficult to understand this evolutionary cessation in the progression of sexual dimorphism and the establishment of sex discrimination in combination with similarities between contest and mating behaviours. However, the recently proposed “erroneous courtship hypothesis” may explain the spontaneous and effective transition from the beginning of contests to courtship behaviour depending on the opponent’s sex^39,40^, which seems to be an economical way to reduce missed opportunity costs. The erroneous courtship hypothesis was first proposed on the basis of courtship behaviour in lycaenid butterflies. Despite the highly differentiated colour patterns between sexes in some species of butterflies, males do not always have the ability to discriminate between sexes. As a result, males of such species always seek females without recognizing sex and often continue mutual chasing if the opponent is male. In this case, the beginnings of chasing and courtship seem to be associated with the same behaviours, leading to the hypothesis that the mutual chasing between two males is caused by erroneous courtship. Two of the proposed prerequisites under this hypothesis are a loss of weaponry with which to attack opponents and an inability to discriminate the opponent’s sex. In our view, the mutual chasing of butterflies resembles the contest behaviour of *S. detrahens*. In the case of *S. detrahens*, individuals do not have powerful weapons when they battle. Instead, in their ritualized contests, they mutually measure their opponent's eye-span, spread their forelegs, and chase opponents that run away. Even in the most intense contests, they undergo only weak head collisions that do not appear to cause physical damage. As a result, *S. detrahens* males are similar to butterfly males that neither have weapons nor distinguish between sexes. Accordingly, the ritualized contests between individuals may share behavioural elements with courtship. If each behaviour is composed of a completely different set of behavioural elements, the missed opportunity cost in courtship behaviour resulting from a random attempt to mate with another individual will increase. Undeveloped sexual dimorphism and sex discrimination associated with similarity between courtship and contest behaviours might be maintained for a long time as an evolutionarily stable strategy.

## Methods

### Stalk-eyed flies

Wild individuals of *S. detrahens* were captured near the shores of several rivers on Ishigaki Island in Okinawa Prefecture (Japan) from June 2012 to November 2019. Adults can be found throughout the year.

### Breeding of *S. detrahens*

To maintain adult flies in the laboratory, disposable plastic vials for *Drosophila* culture or Petri dishes for *Escherichia coli* culture were used. A paper cord soaked with mineral water was provided as a moisture supply, and commercially available semi-dried *Ficus* fruits served as the diet source because the adult individuals in the above wild habitats gather on the dropped fruits of *Ficus variegata* Blume and *Ficus benguetensis* Merrill.

To stimulate the female adults to lay eggs and raise the hatched larvae, large plastic dishes (245 × 245 × 25 mm, Nunc Inc. #240835) with pin holes for aeration were used. In addition to the above-mentioned moisture supply and *Ficus* fruits, dried *Sphagnum* leaves soaked with a certain concentration of royal jelly (v/v) with 0.05% (w/v) butyl parahydroxybenzoate (antimould) served as a provision for the newly hatched larvae. The percentage of royal jelly varied as follows: high-nutrient conditions = 35%, intermediate-nutrient conditions = 20%, and low-nutrient conditions = 5%. Under high- or intermediate-nutrient conditions, the duration of development from the egg to adult stage was approximately 20 days at 25 °C. To move the adult flies in and out of the plastic dishes, carbon dioxide gas was used to anaesthetize them.

### Measurement of external and internal organ sizes

External morphological traits, such as eye span and body length, were measured on the basis of photographs of the flies on 1 mm^2^ paper (for the data in Fig. 2) or by using a VHX-2000 digital microscope (Keyence) (for data other than those in Fig. 2). Eye span was quantified as the linear distance between the left and right outer edges of the compound eyes. With regard to body length, the distance between the frontal tip of the head and the tip of the abdomen was measured in ventral view. Although each eye span and body length was measured only once, measurement error can be neglected because the standard errors of eye span and body length were 0.07% and 0.35%, respectively, when a representative example was measured 20 times with various possible postures.

The sizes of the internal reproductive organs were measured as follows. In the case of the male accessory glands, adult males were maintained for a month to ensure sufficient maturation of the glands. Both the left and right organs were excised together with fine forceps (#5 Dumont) and spring scissors (#15002-08 FST) in phosphate-buffered saline (PBS) (Fig. 3A). After fixation with 4% formalin for more than 20 min, the organs were placed under a coverslip and photographed under a VHX-2000 microscope. The areas of both the left and right accessory glands were measured according to pixel number with ImageJ (NIH). The average area of the two glands was used as the representative value for each individual. In the case of the ovaries, adult females were maintained under the intermediate-nutrient condition (above) for two weeks to ensure sufficient maturation of the ovaries. Both the left and right organs were excised together in PBS (Fig. 3B) as described above for the male accessory glands. After separation of each ovariole from the bilateral ovaries, mature and immature eggs were removed with fine forceps. The immature eggs were identified as small eggs associated with visible nurse cells. The number of mature eggs was used as an index of female reproductive ability. Volume and area were converted to cubic and square roots, respectively, to match dimensions when examining their relationship with eye span (Fig. 3B,C).

### Observation of contests between two individuals

A pair of individuals representing one of the three types of sexual combinations (male vs. male, female vs. female, or male vs. female) was placed in a Petri dish after anaesthesia with carbon dioxide gas. After sufficient awakening, multiple videos, each of which was 17 min in length, were consecutively recorded by a CASIO EX-ZS6 digital camera. The duration of 17 min was due to the functional limitations of this camera, which is sufficiently longer than the time required for fighting behaviour (2–3 s). The cessation of walking in both individuals in a face-to-face orientation was defined as the beginning of a contest. Then, backward or sideways movement was defined as the loser’s motions. Contests composed of fewer than 10 games were excluded in the calculation of winning rates to avoid incidental fluctuation in the winning rate. It was difficult to use the same pair for multiple observations because the frequency of fighting changed day by day.

### Statistics

Student’s t-test, Pearson’s Chi-square test and analysis of covariance (ANCOVA) were carried out by using Excel 2016 MSO (Microsoft Corporation). Linear approximation with model 2 (SMA) regression was carried out by using StatFlex ver. 7 software (Artec Co., Ltd., Osaka, Japan). Sigmoid approximation was performed by the Japan Institute of Statistical Technology by using Minitab 16 software (Minitab Inc., State College, PA, USA). The calculation processes are shown in Supplemental Information 3.

### Mate choice between two individuals of opposite sex

The beginnings of contests and mating behaviour seemed to be indistinguishable (Supplementary Information 1A,B); that is, the cessation of walking of two individuals in a face-to-face orientation was common in the beginning of both contest and mating behaviours, while the subsequent actions were divided into mutual access with the extension of both forelegs in contest behaviour or jumping on the back of the opponent in mating behaviour. Based on this difference, the start of mating could be recognized.

To measure male mate choice, two females with different eye spans and a single male with an intermediate eye span were placed in a Petri dish after anaesthesia with carbon dioxide gas. Males and females can be easily distinguished by observing the external genitalia using a dissection microscope during the period of anaesthesia. After sufficient awakening, time-lapse images were captured for several hours at 30-s intervals with a RICOH GXR digital camera. When checking the time-lapse images, individuals were identified based on differences in eye span. The occurrence of a continuous mounting position for more than 30 s (that is, more than two consecutive images) was defined as successful copulation. Then, the number of copulations with each female was counted.

In the case of female mate choice, experiments were carried out with a male and female combination inverse to that in the experiment described above for male mate choice.

### Experiments to test the ability of males to discriminate flies of different sexes

For Fig. 5A,B: In the first half of the experiment (Step 1), three males with eye spans of different sizes were placed in a Petri dish to induce pseudo-copulation, and the male with an intermediate eye span was the focus when measuring mate choice. Then, in the latter half of the experiment (Step 2), the short-eyed male was replaced with an even shorter-eyed female to continue the measurement of mate choice in the male with the intermediate eye span after awakening from anaesthesia by carbon dioxide.

For Fig. 5C,D: we also tested mate choice among five individuals: long-eyed male/female, short-eyed male/female, and intermediate-eyed male. We focused on mate choice by the intermediate-eyed male and repeated the trial with different combinations of the five individuals.

## Supplementary information

Supplementary Information.
