# Author Correction: Handicap theory is applied to females but not males in relation to mate choice in the stalk-eyed fly *Sphyracephala detrahens*

**DOI:** 10.1038/s41598-023-28547-7

**Published:** 2023-01-26

**Authors:** Koji Takeda, Tomoki Furuta, Masaki Hamada, Yo Sato, Kiichiro Taniguchi, Akihiro Tanizawa, Tomomasa Yagi, Takashi Adachi‑Yamada

**Affiliations:** Department of Life Science, Faculty of Science, Gakushuin University, 1‑5‑1 Mejiro, Toshima‑ku, Tokyo, 171‑8588 Japan

Correction to: *Scientific Reports* 10.1038/s41598-020-76649-3, published online 12 November 2020

The original version of this Article contained errors due to the miscalculation of the winning rates.

Firstly, the data presented in Figure 2 was incorrect. As a result, in panel A, “R^2^ = 0.55” was corrected to “R^2^ = 0.72”; in panel B, “R^2^ = 0.57” was corrected to “R^2^ = 0.74”; and in panel C, “R^2^ = 0.41” was corrected to “R^2^ = 0.61” and “R^2^ = 0.64”.

In panel D, the Table:

**Table:** 

	Eye span	Body length
male-male	0.55	0.35
female-female	0.57	0.46
male–female	0.41	0.29
female-male	0.41	0.29

now reads:

**Table:** 

	Eye span	Body length
male-male	0.72	0.49
female-female	0.74	0.59
male–female	0.64	0.48
female-male	0.61	0.40

As a result, the legend of Figure 2,

“Flies with relatively long eye spans are likely to be winners in contests. Winning rates in 100 [(**A**) male vs. male], 140 [(**B**) female vs. female] and 88 [(**C**) male vs. female] contests including more than 10 games for pairs of randomly chosen individuals with different eye-span lengths were measured and analysed in regard to their relationship with the eye-span ratio (own eye span/opponent’s eye span). Because the rates of both the winner and loser in a contest are simultaneously shown in the same coordinate system, the scatter of the data points shows symmetry around the origin. Thus, to focus on only one player in all contests, ignore values less than 1.0 on the abscissa or less than 0.5 on the ordinate. Only one data point in (**A**), for which the eye-span ratio is 2.15, was excluded as an outlier. Solid lines were drawn by sigmoid approximation via the Gauss–Newton algorithm (Minitab 16, Minitab Inc.). R2 = 0.55 (**A**), 0.57 (**B**), 0.41 [males in (**C**)], and 0.41 [females in (**C**)]. Broken lines indicate 95% confidence intervals. The P values of the three approximation lines are all less than 0.001, indicating significant fits. The P value from the ANCOVA of the datum point distribution among the three kinds of sexual combinations was 0.9998, indicating a non-significant difference.”

now reads:

“Flies with relatively long eye spans are likely to be winners in contests. Winning rates in 100 (**A**, male vs. male), 140 (**B**, female vs. female) and 88 (**C**, male vs. female) contests including more than 10 games for pairs of randomly chosen individuals with different eye-span lengths were measured and analysed in regard to their relationship with the eye-span ratio (own eye span/opponent’s eye span). Each datum point represents one player’s outcome in a contest on one pair of flies with different eye spans. A single contest generates two data points: one for long-eyed fly and the other for short-eyed fly. Because all the winning rates included draws in the denominator, the mean values in each sexual combination are less than 0.5. Only one data point in A, for which the eye-span ratio is 2.15, was excluded as an outlier. Solid lines were drawn by sigmoid approximation via the Gauss-Newton algorithm (Minitab 16, Minitab Inc.). R^2^ = 0.72 (**A**), 0.74 (**B**), 0.64 (males in **C**), and 0.61 (females in **C**). Broken lines indicate 95% confidence intervals. The P values of the three approximation lines are all less than 0.001, indicating significant fits. The P value from the ANCOVA of the datum point distribution among the three kinds of sexual combinations was 0.9469, indicating a non-significant difference.”

The original Figure 2 and accompanying legend appear below.

**Figure 2:**
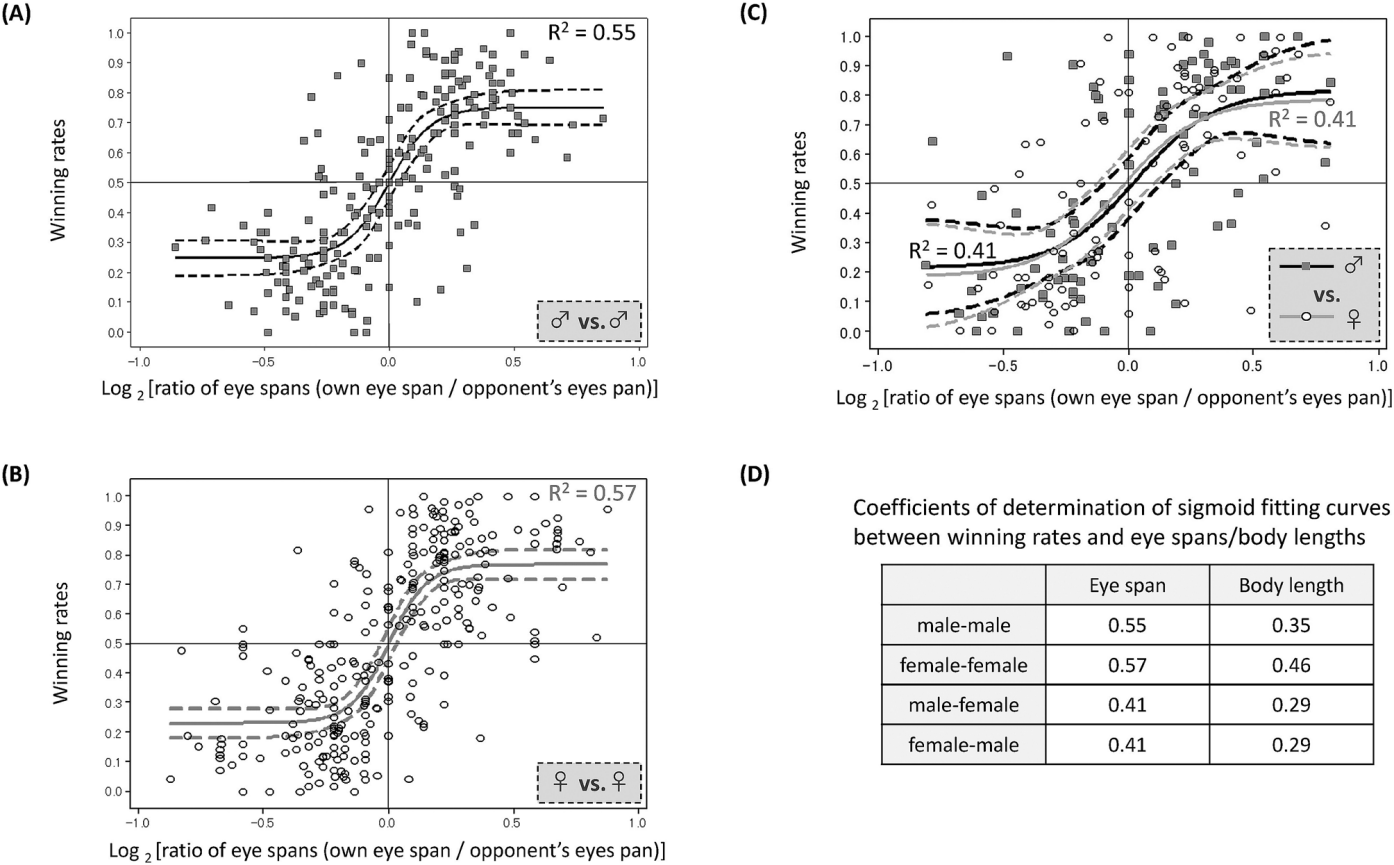
Flies with relatively long eye spans are likely to be winners in contests. Winning rates in 100 [(**A**) male vs. male], 140 [(**B**) female vs. female] and 88 [(**C**) male vs. female] contests including more than 10 games for pairs of randomly chosen individuals with different eye-span lengths were measured and analysed in regard to their relationship with the eye-span ratio (own eye span/opponent’s eye span). Because the rates of both the winner and loser in a contest are simultaneously shown in the same coordinate system, the scatter of the data points shows symmetry around the origin. Thus, to focus on only one player in all contests, ignore values less than 1.0 on the abscissa or less than 0.5 on the ordinate. Only one data point in (**A**), for which the eye-span ratio is 2.15, was excluded as an outlier. Solid lines were drawn by sigmoid approximation via the Gauss–Newton algorithm (Minitab 16, Minitab Inc.). R^2^ = 0.55 (**A**), 0.57 (**B**), 0.41 [males in (**C**)], and 0.41 [females in (**C**)]. Broken lines indicate 95% confidence intervals. The P values of the three approximation lines are all less than 0.001, indicating significant fits. The P value from the ANCOVA of the datum point distribution among the three kinds of sexual combinations was 0.9998, indicating a non-significant difference.

Consequently, the values present in the Results section, under the subheading ‘Hypothesis 1: Eye span as an honest signal to inform opponents of fighting capacity’, were incorrect. As a result,

“The coefficient of determination representing the correlation was 0.55. Similar striking correlations were also found in contests between females (Fig. 2B) and in contests between males and females (Fig. 2C), with winning rates for longer-eyed individuals of 85% (P value = 6.3 × 10^–18^) in female-female contents and 78% (P value = 5.4 × 10^–8^) in male–female contents and coefficients of determination of sigmoid fitting curves of 0.57 and 0.41, respectively. In the contests between males and females, neither sex was more likely to win. The fitted curves for these three kinds of sexual combinations were very similar and indistinguishable by covariance analysis. Based on these results, it appears that this species participates in contests in which eye span acts as an honest signal of fighting capacity that informs the opponent similarly in all three kinds of sexual combinations. However, among the contests in which the difference between the eye spans of both individuals was small (less than 10%), the male-male contest displaying the winning rate of around 50% (more than 40% and less than 60%) showed a higher percentage than contests of other sexual combinations (32% in male-male contents, 16% in female-female contests, and 10% in male–female contests). This might suggest a tendency for males to not readily give up during a game.”

now reads:

“The coefficient of determination representing the correlation was 0.72. Similar striking correlations were also found in contests between females (Fig. 2B) and in contests between males and females (Fig. 2C), with winning rates for longer-eyed individuals of 85% (P value = 6.3 × 10^–18^) in female-female contents and 78% (P value = 5.4 × 10^–8^) in male–female contents and coefficients of determination of sigmoid fitting curves of 0.74 and 0.61–0.64, respectively. In the contests between males and females, neither sex was more likely to win. The fitted curves for these three kinds of sexual combinations were very similar and indistinguishable by covariance analysis. Based on these results, it appears that this species participates in contests in which eye span acts as an honest signal of fighting capacity that informs the opponent similarly in all three kinds of sexual combinations. However, among the contests in which the difference between the eye spans of both individuals was small (less than 10%), the male-male contest displaying the winning rate of around average 41.9% (more than 31.9% and less than 51.9%) showed a higher proportion than contests of other sexual combinations (26% in male-male contents, 13% in female-female contests, and 14% in male–female contests). This might suggest a tendency for males to not readily give up during a game.”

In addition, the data in Tables ‘Estimated values of parameters’ and ‘Coefficient of determination’, present in the Supplementary Information, Section 3: ‘Statistical method for fitting sigmoid curves to the relationship between winning rate and eye-span ratio between players’ was incorrect. The correct and incorrect values appear below. The original Supplementary Information is provided below.

Incorrect:

Estimated values of parameters:

**Table:** 

	Regression coefficient	Estimate	Standard error	95% Confidence intervals
Male vs male	Theta1	0.752089	0.0297833	(0.697686, 0.828404)
	Theta2	0.247911	0.0297833	(0.171596, 0.302314)
	Theta3	− 0.000000	0.0254107	(− 0.052998, 0.052998)
	Theta4	0.083989	0.0234799	(0.042551, 0.153038)
Female vs female	Theta1	0.769293	0.0257683	(0.722399, 0.825377)
	Theta2	0.230707	0.0257683	(0.174623, 0.277601)
	Theta3	0.000000	0.0205945	(− 0.041031, 0.041031)
	Theta4	0.076302	0.0172004	(0.047411, 0.116102)
Males in male vs female	Theta1	0.816198	0.0978977	(*, 1.35182)
	Theta2	0.214554	0.0869185	(‐0.146609, 0.34818)
	Theta3	0.032999	0.0924641	(− 0.168222, 0.33956)
	Theta4	0.156657	0.0819604	(0.056978, 0.57010)
Females in male vs female	Theta1	0.785446	0.0869185	(0.651821, 1.14661)
	Theta2	0.183802	0.0978977	(− 0.351816, *)
	Theta3	− 0.032999	0.0924640	(− 0.339557, 0.16822)
	Theta4	0.156657	0.0819603	(0.056978, 0.57010)

Coefficient of determination:

**Table:** 

	Factors	DF	Square sum	Mean square	F-value	p-value
Male vs male	Regression	1	8.3362	8.3362	246.27	< 0.001
	Residual error	198	6.7022	0.0338		
	Sum	199	15.0384			
	Coefficient of determination: 8.3362/15.0384 = 0.554					
Female vs female	Regression	1	13.971	13.971	372.46	< 0.001
	Residual error	278	10.428	0.038		
	Sum	279	24.399			
	Coefficient of determination: 13.971/24.399 = 0.573					
Males in male vs female	Regression	1	7.111	3.9105	60.24	< 0.001
	Residual error	86	4.056	0.0649		
	Sum	87	11.167			
	Coefficient of determination: 3.9105/9.4934 = 0.412					
Females in male vs female	Regression	1	3.9105	3.9105	60.24	< 0.001
	Residual error	86	5.5828	0.0649		
	Sum	87	9.4934			
	Coefficient of determination: 3.9105/9.4934 = 0.412					

Correct:

Estimated values of parameters:

**Table:** 

	Regression coefficient	Estimate	Standard error	95% Confidence intervals
Male vs male	Theta1	0.740116	0.0220877	(0.697420, 0.788496)
	Theta2	0.133642	0.0215938	(0.087323, 0.175114)
	Theta3	0.006398	0.0132290	(− 0.019918, 0.033322)
	Theta4	0.055613	0.0120333	(0.032345, 0.087582)
Female vs female	Theta1	0.759356	0.0197601	(0.721084, 0.801000)
	Theta2	0.107508	0.0190800	(0.068658, 0.143590)
	Theta3	0.009126	0.0114918	(− 0.013435, 0.032022)
	Theta4	0.052528	0.0093982	(0.034393, 0.073573)
Males in male vs female	Theta1	0.801408	0.0611882	(*, 0.943666)
	Theta2	0.089077	0.0542438	(− 0.0277675, 0.183084)
	Theta3	0.051497	0.0439795	(− 0.0402507, 0.139824)
	Theta4	0.106692	0.0368689	(0.0566858, 0.188948)
Females in male vs female	Theta1	0.788780	0.0673040	(*, 0.939862)
	Theta2	0.048852	0.0673549	(− 0.123703, 0.161645)
	Theta3	− 0.008878	0.0525570	(− 0.121066, 0.095168)
	Theta4	0.127686	0.0439338	(0.069573, 0.237555)

Coefficient of determination:

**Table:** 

	Factors	DF	Square sum	Mean square	F-value	p-value
Male vs male	Regression	1	14.079	14.0795	518.92	< 0.001
	Residual error	198	5.372	0.0271		
	Sum	199	19.452			
	Coefficient of determination: 14.0795/19.452 = 0.724					
Female vs female	Regression	1	23.568	23.5684	777.13	< 0.001
	Residual error	278	8.431	0.0303		
	Sum	279	31.999			
	Coefficient of determination: 23.5684/31.999 = 0.737					
Males in male vs female	Regression	1	7.111	7.11071	150.78	< 0.001
	Residual error	86	4.056	0.04716		
	Sum	87	11.167			
	Coefficient of determination: 7.11071/11.167 = 0.637					
Females in male vs female	Regression	1	6.904	6.90449	136.56	< 0.001
	Residual error	86	4.348	0.05056		
	Sum	87	11.253			
	Coefficient of determination: 6.90449/11.253 = 0.614					

The original Article and accompanying Supplementary Information file have been corrected.

## Supplementary Information

Supplementary Information.

