# Supplementary material for: Handicap theory is applied to females but not males in relation to mate choice in the stalk-eyed fly Sphyracephala detrahens
Source: Sci Rep. 2020 Nov 12;10:19684. doi: 10.1038/s41598-020-76649-3 (PMC7661502; doi:10.1038/s41598-020-76649-3)
Supplement: Supplementary file 1 — Supplementary Information. [file 41598_2020_76649_MOESM1_ESM.pdf]

Supplementary information (Correction)

Handicap theory is applied to females but not males in mate choice in the stalk-eyed fly *Sphyracephala detrahens*

Koji Takeda, Tomoki Furuta*, Masaki Hamada*, Yo Sato*, Kiichiro Taniguchi*, Akihiro Tanizawa*, Tomomasa Yagi*, and Takashi Adachi-Yamada**

Department of Life Science, Faculty of Science, Gakushuin University, 1-5-1 Mejiro, Toshima-ku, Tokyo 171-8588, Japan

*Authors except for those listed first and last equally contributed to this work and are shown in alphabetical order of the last names.

****Contact information**: Takashi Adachi-Yamada
Phone: +81-3-5904-9411
Fax: +81-3-5992-1029
E-mail address: Takashi.Adachi-Yamada@gakushuin.ac.jp

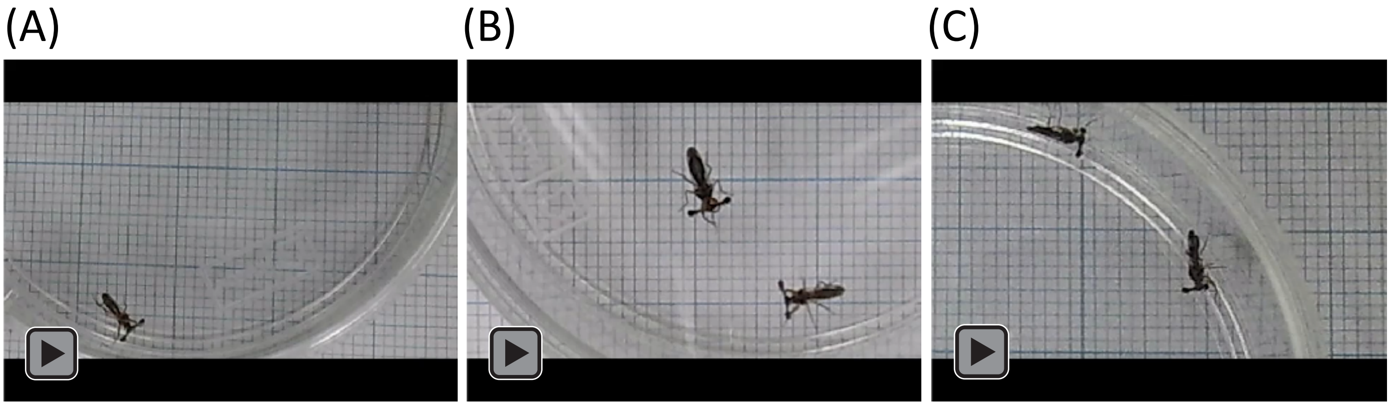

Supplementary information 1.
Videos of contests, courtship, and pseudocopulation in *S. detrahens*.

(A) Contest between two males.
(B) Courtship between a male and a female.
(C) Pseudocopulation between two males.

Gridline intervals are 1 mm in all videos.

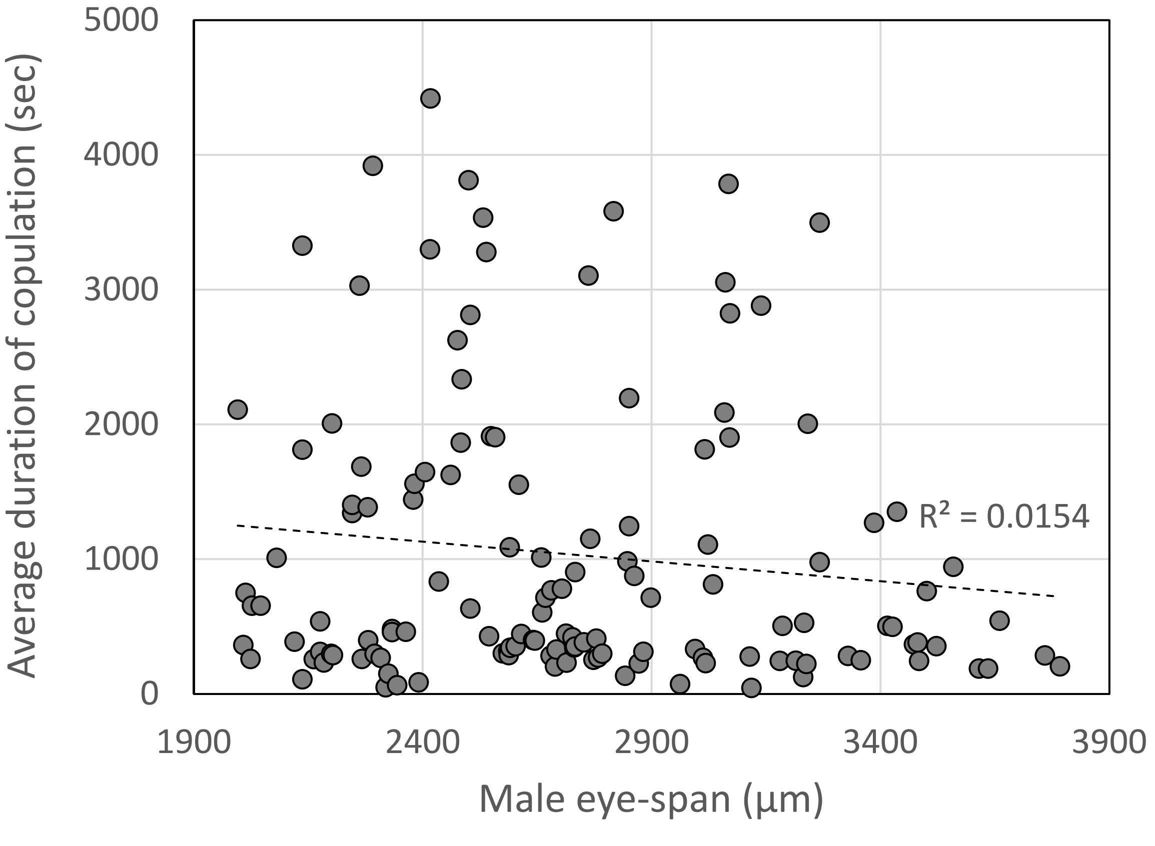

Supplementary information 2.
The duration of copulation is unrelated to eye-span in males.

Fitting curve: Winning rates vs binary logarithm of eye-span ratio
Non-linear (sigmoid) regression formula:
Winning rate = Theta1 + (Theta2 - Theta1) / (1 + exp((binary logarithm of size ratio - Theta3) / Theta4))
Method: Gauss-Newton algorithm

Estimated values of parameters:

	Regression coefficient	Estimate	Standard error	95 % Confidence intervals
male vs male	Theta1	0.740116	0.0220877	(0.697420, 0.788496)
	Theta2	0.133642	0.0215938	(0.087323, 0.175114)
	Theta3	0.006398	0.0132290	(-0.019918, 0.033322)
	Theta4	0.055613	0.0120333	(0.032345, 0.087582)
female vs female	Theta1	0.759356	0.0197601	(0.721084, 0.801000)
	Theta2	0.107508	0.0190800	(0.068658, 0.143590)
	Theta3	0.009126	0.0114918	(-0.013435, 0.032022)
	Theta4	0.052528	0.0093982	(0.034393, 0.073573)
males in male vs female	Theta1	0.801408	0.0611882	(*, 0.943666)
	Theta2	0.089077	0.0542438	(-0.0277675, 0.183084)
	Theta3	0.051497	0.0439795	(-0.0402507, 0.139824)
	Theta4	0.106692	0.0368689	(0.0566858, 0.188948)
females in male vs female	Theta1	0.788780	0.0673040	(*, 0.939862)
	Theta2	0.048852	0.0673549	(-0.123703, 0.161645)
	Theta3	-0.008878	0.0525570	(-0.121066, 0.095168)
	Theta4	0.127686	0.0439338	(0.069573, 0.237555)

Coefficient of determination:

	Factors	DF	Square sum	Mean square	F-value	p-value
male vs male	Regression	1	14.079	14.0795	518.92	<0.001
	Residual error	198	5.372	0.0271		
	Sum	199	19.452			
	Coefficient of determination: 14.0795/19.452 = 0.724					
female vs female	Regression	1	23.568	23.5684	777.13	<0.001
	Residual error	278	8.431	0.0303		
	Sum	279	31.999			
	Coefficient of determination: 23.5684/31.999 = 0.737					
males in male vs female	Regression	1	7.111	7.11071	150.78	<0.001
	Residual error	86	4.056	0.04716		
	Sum	87	11.167			
	Coefficient of determination: 7.11071/11.167 = 0.637					
females in male vs female	Regression	1	6.904	6.90449	136.56	<0.001
	Residual error	86	4.348	0.05056		
	Sum	87	11.253			
	Coefficient of determination: 6.90449/11.253 = 0.614					

Supplementary information 3 (Correction).
Statistical method for fitting sigmoid curves to the relationship between winning rate and eye-span ratio between players.

We corrected the data values in the calculation process of the sigmoid fitting curves as above according to the correction of the calculation method of the winning rates.

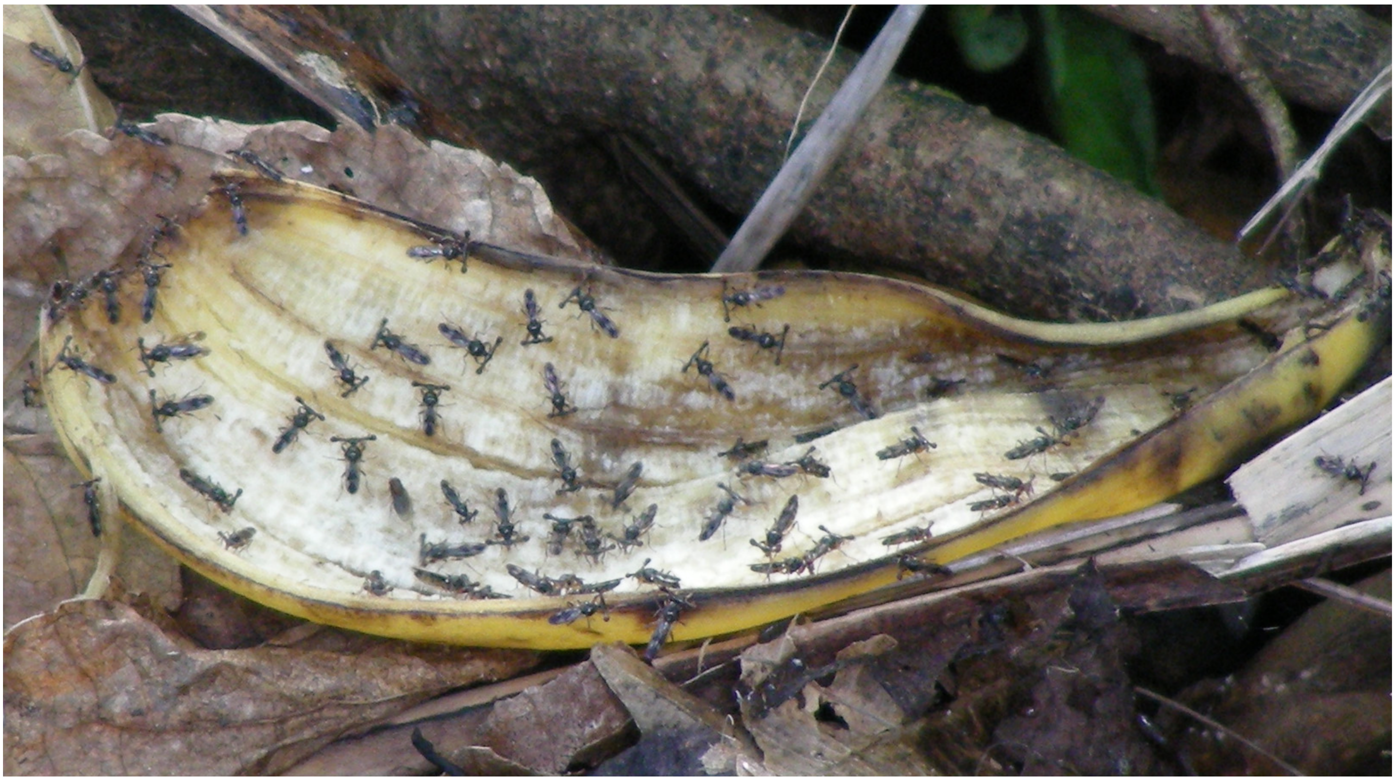

Supplementary information 4.
Gathering of adults of *S. detrahens* to an artificially supplied skin of *Musa* (banana) fruit in natural habitat (Ishigaki Is., Japan).

The adults do not show severe fighting under such nutrient-rich conditions.
