# Supplementary material for: Author Correction: Handicap theory is applied to females but not males in relation to mate choice in the stalk-eyed fly Sphyracephala detrahens
Source: Sci Rep. 2023 Jan 26;13:1463. doi: 10.1038/s41598-023-28547-7 (PMC9879937; doi:10.1038/s41598-023-28547-7)
Supplement: Supplementary file 1 — Supplementary Information. [file 41598_2023_28547_MOESM1_ESM.pdf]

Supplementary information

Handicap theory is applied to females but not males in mate choice in the stalk-eyed fly *Sphyracephala detrahens*

Koji Takeda, Tomoki Furuta*, Masaki Hamada*, Yo Sato*, Kiichiro Taniguchi*, Akihiro Tanizawa*, Tomomasa Yagi*, and Takashi Adachi-Yamada**

Department of Life Science, Faculty of Science, Gakushuin University, 1-5-1 Mejiro, Toshima-ku, Tokyo 171-8588, Japan

*Authors except for those listed first and last equally contributed to this work and are shown in alphabetical order of the last names.

****Contact information**: Takashi Adachi-Yamada
Phone: +81-3-5904-9411
Fax: +81-3-5992-1029
E-mail address: Takashi.Adachi-Yamada@gakushuin.ac.jp

(A) (B) (C)

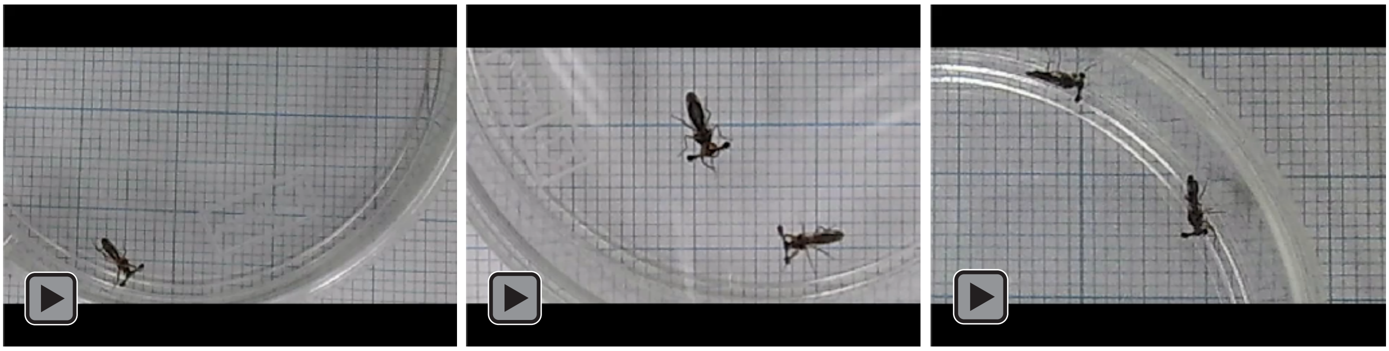

Supplementary information 1.
Videos of contests, courtship, and pseudocopulation in *S. detrahens*.

(A) Contest between two males.
(B) Courtship between a male and a female.
(C) Pseudocopulation between two males.

Gridline intervals are 1 mm in all videos.

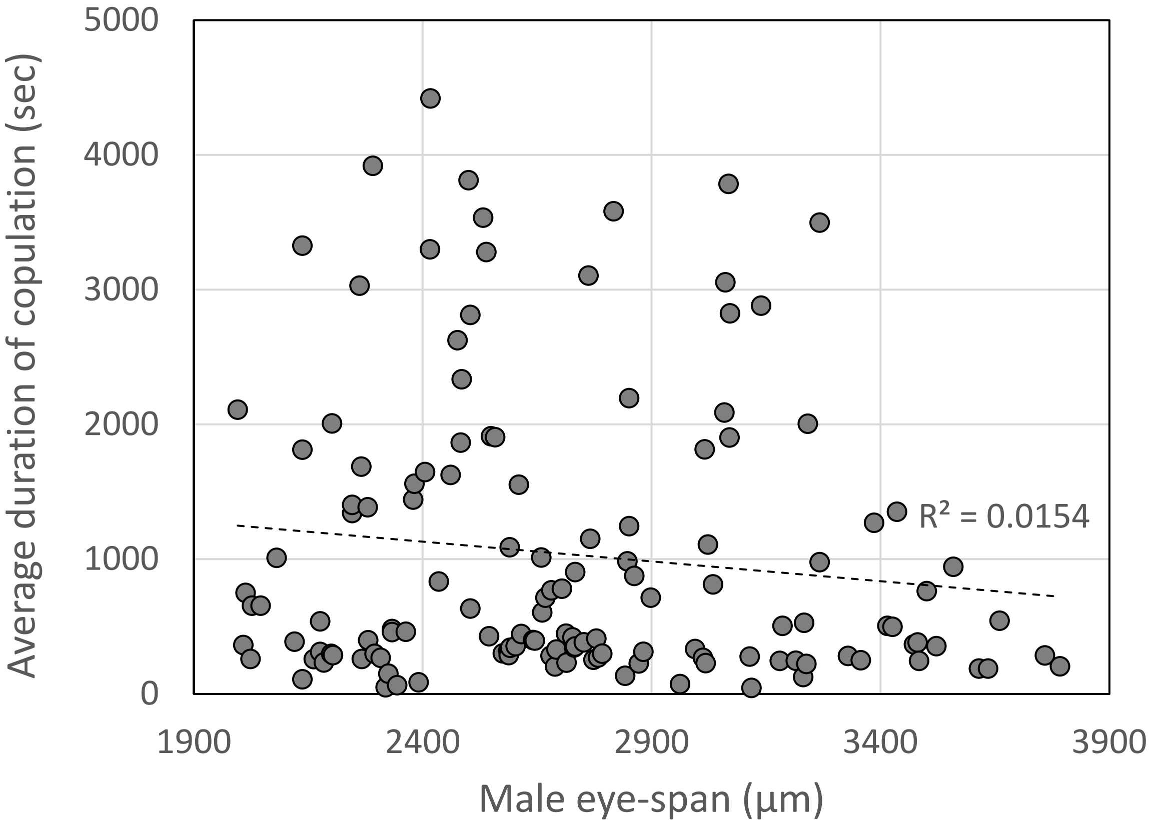

Supplementary information 2.
The duration of copulation is unrelated to eye-span in males.

Fitting curve: Winning rates vs binary logarithm of eye-span ratio
Non-linear (sigmoid) regression formula:
Winning rate = Theta1 + (Theta2 - Theta1) / (1 + exp((binary logarithm of size ratio - Theta3) / Theta4))
Method: Gauss-Newton algorithm

Estimated values of parameters:

	Regression coefficient	Estimate	Standard error	95 % Confidence intervals
male vs male	Theta1	0.752089	0.0297833	(0.697686, 0.828404)
	Theta2	0.247911	0.0297833	(0.171596, 0.302314)
	Theta3	-0.000000	0.0254107	(-0.052998, 0.052998)
	Theta4	0.083989	0.0234799	(0.042551, 0.153038)
female vs female	Theta1	0.769293	0.0257683	(0.722399, 0.825377)
	Theta2	0.230707	0.0257683	(0.174623, 0.277601)
	Theta3	0.000000	0.0205945	(-0.041031, 0.041031)
	Theta4	0.076302	0.0172004	(0.047411, 0.116102)
males in male vs female	Theta1	0.816198	0.0978977	(*, 1.35182)
	Theta2	0.214554	0.0869185	(-0.146609, 0.34818)
	Theta3	0.032999	0.0924641	(-0.168222, 0.33956)
	Theta4	0.156657	0.0819604	(0.056978, 0.57010)
females in male vs female	Theta1	0.785446	0.0869185	(0.651821, 1.14661)
	Theta2	0.183802	0.0978977	(-0.351816, *)
	Theta3	-0.032999	0.0924640	(-0.339557, 0.16822)
	Theta4	0.156657	0.0819603	(0.056978, 0.57010)

Coefficient of determination:

	Factors	DF	Square sum	Mean square	F-value	p-value
male vs male	Regression	1	8.3362	8.3362	246.27	<0.001
	Residual error	198	6.7022	0.0338		
	Sum	199	15.0384			
	Coefficient of determination: 8.3362/15.0384 = 0.554					
female vs female	Regression	1	13.971	13.971	372.46	<0.001
	Residual error	278	10.428	0.038		
	Sum	279	24.399			
	Coefficient of determination: 13.971/24.399 = 0.573					
males in male vs female	Regression	1	3.9105	3.9105	60.24	<0.001
	Residual error	86	5.5828	0.0649		
	Sum	87	9.4934			
	Coefficient of determination: 3.9105/9.4934 = 0.412					
females in male vs female	Regression	1	3.9105	3.9105	60.24	<0.001
	Residual error	86	5.5828	0.0649		
	Sum	87	9.4934			
	Coefficient of determination: 3.9105/9.4934 = 0.412					

Supplementary information 3.
Statistical method for fitting sigmoid curves to the relationship between winning rate and eye-span ratio between players.

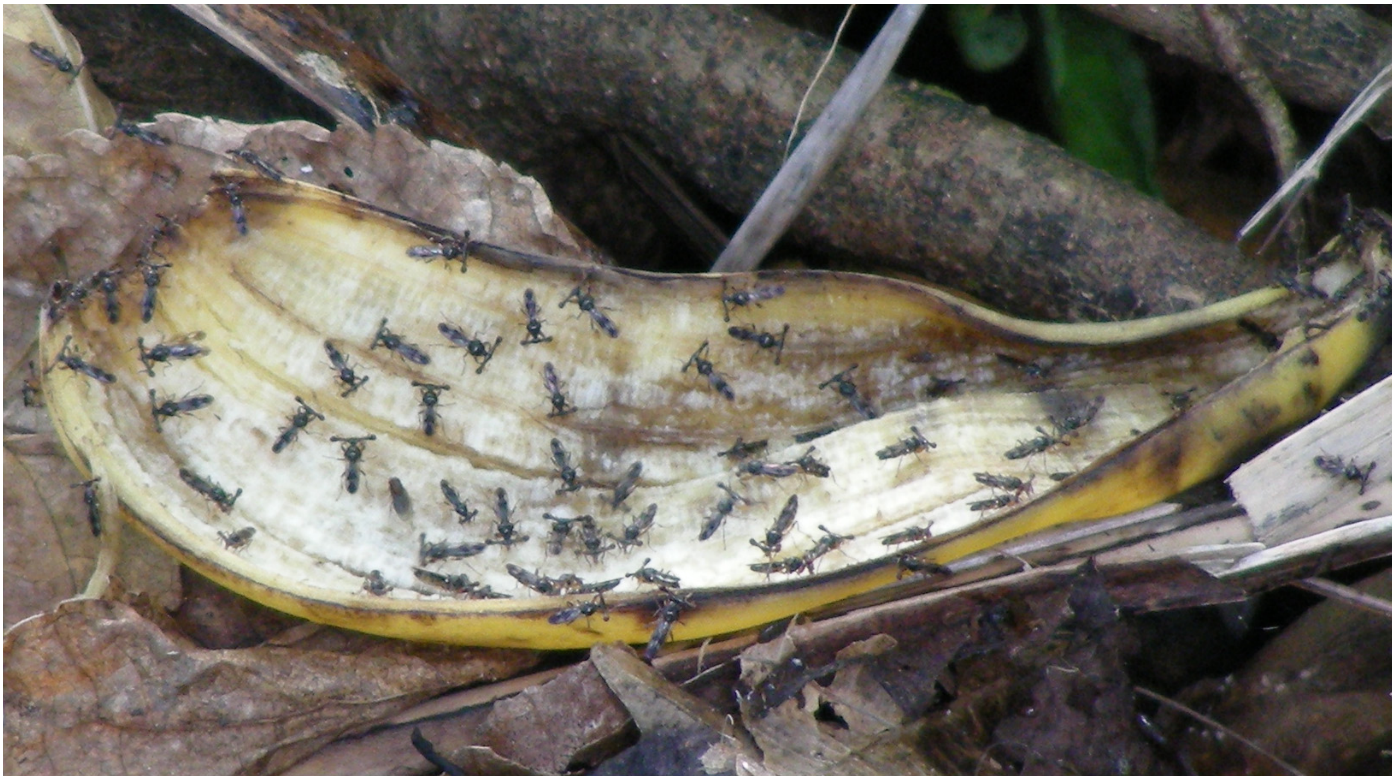

Supplementary information 4.
Gathering of adults of *S. detrahens* to an artificially supplied skin of *Musa* (banana) fruit in natural habitat (Ishigaki Is., Japan).

The adults do not show severe fighting under such nutrient-rich conditions.
